# Quantification of Sound Exposure from Wind Turbines in France

**DOI:** 10.3390/ijerph19010023

**Published:** 2021-12-21

**Authors:** David Ecotière, Patrick Demizieux, Gwenaël Guillaume, Lise Giorgis-Allemand, Anne-Sophie Evrard

**Affiliations:** 1 UMRAE, Cerema, Univ Gustave Eiffel, IFSTTAR, F-67035 Strasbourg, France; david.ecotiere@cerema.fr (D.E.); patrick.demizieux@laposte.net (P.D.); gwenael.guillaume@cerema.fr (G.G.); 2 Umrestte UMR T9405, Univ Lyon, Univ Gustave Eiffel, IFSTTAR, Univ Lyon 1, F-69675 Bron, France; lise.giorgis-allemand@univ-eiffel.fr

**Keywords:** wind turbine, sound exposure, environmental noise, public health

## Abstract

The WHO guidelines on environmental noise highlight that evidence on the health effects of wind turbine sound levels is either non-existent or of poor quality. In this context, a feasibility study was conducted in France in 2017. The objective was to suggest a methodology for calculating wind turbine sound levels in order to quantify the number of windfarms’ residents exposed to this sound. Based on a literature review, the Harmonoise model was selected for sound exposure calculation. It was validated by quantifying its uncertainties, and finally used to estimate the population exposed to wind turbine sound in metropolitan France. Compared to other environmental noise sources (e.g., transportation), sound exposure is very moderate, with more than 80% of the exposed people exposed to sound levels below 40 dBA. The total number of people exposed to more than 30 dBA is about 686,000 and 722,000 people for typical daytime and night-time meteorological conditions respectively, i.e., about 1% of the French population in 2017. These results represent the first ever assessment of sound exposure from wind turbines at the scale of the entire metropolitan France.

## 1. Introduction

Wind energy is expanding rapidly in France, as it is everywhere else in the world. Specific rules govern the design and implementation of wind farms in order to limit the noise they produce when in operation. However, the population living near these installations is often concerned about the health impacts of sound levels emitted by wind turbines (WT) and there is a lack of available scientific data on this topic.

Most studies have found a significant positive association between WT sound levels and the percentage of highly annoyed people [1,2]. Very few studies have investigated the effects of WT sound on sleep disturbance, cardiovascular disease, effects on metabolic or endocrine systems, or on cognition or mental health. Therefore, the WHO guidelines on environmental noise published in October 2018 pointed out that the evidence on the health effects of wind turbines noise is either non-existent or of low quality [3]. The WHO and Anses (French Agency for Food, Environmental and Occupational Health & Safety) in France therefore recommended implementing epidemiological studies [3,4,5]. However, a number of issues remained to be overcome before such a study can be carried out in France.

The first issue concerned the estimation of exposure to WT sound. Indeed, the quality of epidemiological studies evaluating the risks related to environmental exposures depends in part on the quality of the estimation or measurement of the participants’ exposure. However, there was no real consensus on a WT sound prediction model and it was necessary to provide validation criteria to identify the most relevant model.

The second issue concerned the count of the number of people exposed to WT sound. Unlike other sources of noise pollution (e.g., transportation noise), wind farms are generally built in sparsely populated areas, and consequently the number of people potentially exposed to WT sound seemed a priori to be much smaller than the number of residents exposed to other sources of anthropogenic noise, such as transportation noise, for example. In order to conduct an epidemiological study, it would be necessary to recruit a sufficient number of individuals exposed to different and relatively contrasting WT sound levels. Thus, it was necessary to be able to estimate the population exposed to various WT sound levels at the scale of an entire country. This had never been done before in France.

In this context, a feasibility study for an epidemiological study called Cibélius (Knowing the impact of wind turbine noise on health) was conducted in France between 2017 and 2019. The objective was to propose a methodology for calculating WT sound levels and to identify the number of French residents exposed to different sound levels of wind turbine.

The aim of the research presented in this paper was to quantify the number of windfarms’ residents in France exposed to audible WT sound. For this purpose, a WT sound prediction model was selected and validated for the calculation of sound exposure. Then a methodology was suggested for estimating the number of people exposed to WT sound at the scale of all metropolitan France. A brief comparison with transportation noise exposure was also investigated.

## 2. Material and Methods

### 2.1. Overview of the Methodology

As it would not be feasible to make sound levels measurements in all the dwellings of people exposed to WT sound at the scale of the whole French national territory, it was necessary to estimate sound exposure by using an appropriate numerical modeling of WT sound emission and propagation. We first presented the numerical model used, and its performances for WT sound prediction by quantifying its uncertainties. Then, we detailed the method of calculation of sound exposure at the scale of the metropolitan French territory. Finally, the method for evaluating the count of people exposed to WT sound was shown.

### 2.2. Selection and Validation of a Numerical Wind Turbine Sound Model

The sound levels prediction model was selected from a literature review based on the following essential criteria in the context of WT sound: ability to account for a high noise source (hub height above 60 m), topography properties, meteorological (vertical profiles of wind speed, wind direction and temperature) and ground effects on sound propagation. The model must also be able to parameterize the sound power of the source as a function of wind speed. Although some research propagation models could meet these criteria for WT sound [6,7,8,9], they were not suitable for sound levels modeling on a scale as large as a country’s territory, because of their high computational time.

Although it was less frequently used [10,11] than other engineering models that can handle a large-scale territory (e.g., ISO 9613-2 [12], NMPB-2008 [13] or CNOSSOS-EU [14]), the Harmonoise model [15] was preferred here for WT sound prediction because it takes into account ground effects more accurately, and because it is able to model the effects of different wind speeds and directions, and different classes of atmospheric stability on sound propagation. These properties are essential for the modeling of WT sound propagation, and several authors have mentioned the capabilities of this model for WT sound predictions [16,17,18].

The uncertainties of the Harmonoise model were estimated by comparison between numerical modeling and in situ measurements. For this purpose, an experimental campaign was carried out near a wind farm whose characteristics were representative of the vast majority of French wind farms: flat site (terrain slope lower than 2% over 2000 m), quiet environment, good diversity of wind speeds and directions. The wind farm consisted of five wind turbines with a rated electrical power of 2 MW each. Each WT had a hub located at 100 m high, and three 46 m long blades. During the eight days of the measurement period, the wind farm operated in 1-h /1-h on/off cycles, in order to select only measurements with a satisfactory signal to noise ratio between WT sound and background noise of the site, and thus not to retain sound samples containing extraneous noise.

**Figure 1:**
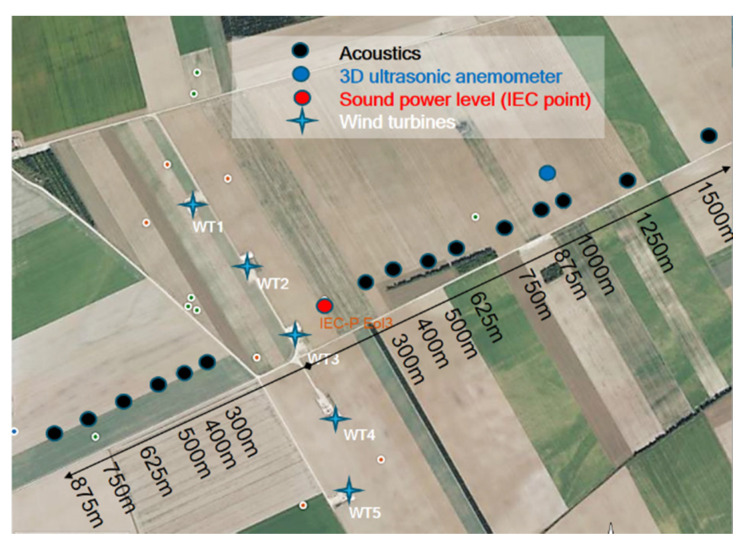
General overview of the experimental set-up for the validation of the numerical model. Location of sound level measurements (black points), wind measurements (blue points), wind turbine sound power level measurements (red points), wind turbines (blue crosses).

LAeq (10 min) sound levels were recorded using 15 sound level meters located on the two dominant wind directions of the site, and at distances from the wind farm ranging from 0 to 1500 m (Figure 1). This arrangement enabled sound levels to be measured for downwind and upwind situations, for which sound propagation differs [19]. The sound level meters (B&K 2250, ACOEM Solo and Cube, Rion NL62) were placed at a height of 1.5 ± 0.1 m above the ground and measured in the frequency bands [12.5 Hz; 20 kHz] or [1 Hz; 20 kHz] depending on the point. In addition to these sound level measurements, the sound level power of the wind turbines was derived according to the protocol described in the standard IEC 61400-11 [20], based on sound levels measurements made with a flush-mounted microphone on a circular reflective plate of 1 m diameter, placed on the ground 143 m from a wind turbine (Figure 1).

Meteorological measurements were made in order to obtain the influence of wind speed and direction on both emission and propagation of WT sound. Wind speed and direction measured from a 3D ultrasonic anemometer (Campbell CSAT3) located at 3 m high were used to classify sound propagation conditions as required by the Harmonoise modeling [10]. Wind speed at hub height and wind turbine operation data (speed rotation of the rotor, electrical production) were obtained from the wind farm’s Supervisory Control And Data Acquisition system (SCADA). The accuracy of the anemometers (of the Campbell device or the WT hub) was typically 0.1 m/s, which was satisfactory for not inducing a significant uncertainty in the wind speed classification required for the analysis.

The WT emission was modeled using the manufacturer’s WT technical specifications, which give the sound power level as a function of wind speed. An additional 2.2 dBA was added to these values to match the actual sound power level measured at the IEC point. The uncertainties of the sound levels prediction model were estimated by calculating the distribution of the deviation between the predicted and measured sound levels. The bias was estimated with the mean of this distribution, and the standard uncertainty with its standard deviation [21]. The hypothesis adopted for the calculation process of WT sound assumed WT operating at full power (see Section 2.3.2). To be in line with this hypothesis, only periods when wind speed was above 6 m/s at 10 m high were kept for the calculation of the bias and the standard uncertainty. Similarly, in order to be consistent with French regulations that do not allow wind farms to be installed less than 500 m from local residents, only measurements for which the distance to the wind farm was greater than 500 m were kept. Bias was used to correct the sound levels predicted by the Harmonoise model, while the standard uncertainty was used to bound the estimate of the population exposed to WT sound and to account for the uncertainty in the Harmonoise model’s estimate of sound levels on the count of the exposed population. Indeed, the population count was performed by considering three scenarii of sound exposure: the first one (average scenario) used the sound levels predicted by the Harmonoise model, only corrected by the estimated bias, and the other two scenarii (upper and lower scenarii) used the sound levels predicted by the model, corrected by the bias, and increased or decreased respectively by a standard uncertainty in the sound level estimate. It should be noted that the range given by the uncertainty estimates based on the lower and upper scenarii did not correspond to confidence intervals associated with a level of reliability. They did, however, provide information on the best and worst-case values.

As meteorological conditions (wind and temperature vertical profiles) could have significant effects on long-range propagation of outdoor sound, resulting in decreased or increased noise levels at dwellings, it was essential to separate daytime and night-time periods where the meteorological influence on propagation often differs [19]. The uncertainties were thus calculated for two propagation conditions representative of daytime and night-time meteorological conditions.

### 2.3. Estimation of Sound Exposure from Wind Turbines

The selected sound levels prediction model was used to produce a noise map of all the wind farms on the metropolitan French territory. This mapping was built following the three steps detailed below.

#### 2.3.1. Constitution of a Database with the Characteristics Required for the Prediction of Sound Levels from Wind Farms in Metropolitan FRANCE

**Figure 2:**
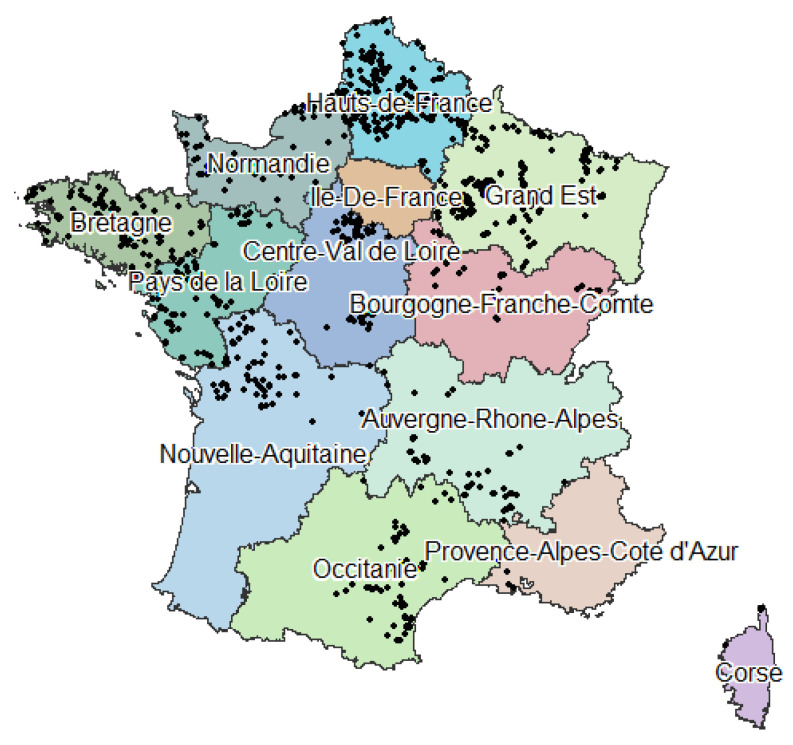
Localisation of metropolitan French wind farms (2017, www.thewindpower.net, accessed on 1 December 2021).

The location of the wind farms (Figure 2), the hub height and the rated electrical power of the wind turbines were obtained from a 2017 database provided by www.thewindpower.net (accessed on 1 December 2021), which was the most complete database publicly available at the beginning of this research. This database listed existing wind farms as of 30 August 2017, of which only wind farms in operation in metropolitan France were considered here. The absolute position of each WT within each wind farm was obtained from the BDTOPO^®^ topographic database [22] from the National Institute of Geographic and Forestry Information (IGN).

#### 2.3.2. Calculation of Sound Contributions of Wind Turbines near the Dwellings of Wind Farm Residents

Sound levels calculations with the Harmonoise model presented earlier were performed with the environmental noise prediction software CadnaA from DataKustik [23]. In this software, each wind turbine was modeled as a point source located at the height of the wind turbine hub. Following the results of Botha et al. [24], Møller et al. [25] and Tachibana et al. [26], the sound emission of each wind turbine was estimated considering a linear sound emission spectrum (−4 dB/octave), whose overall sound power level was estimated from its rated electrical power [25], considering a wind speed of 7 m/s at 10 m height (nominal operation of the wind turbines in full operation).

**Figure 3:**
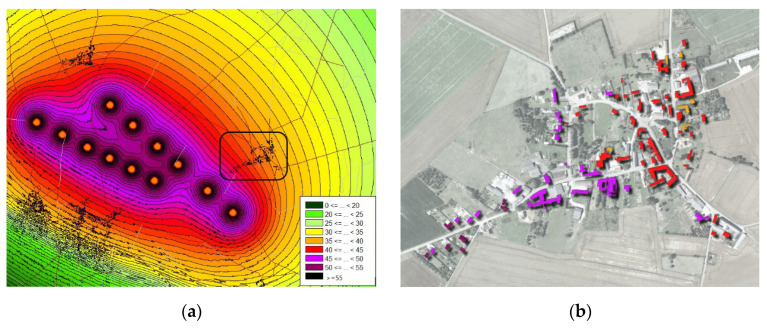
Example of the result of the calculation of the radiated sound from a wind farm (**a**), and of the exposure of the buildings in the nearby village enclosed by the black block in (**a**,**b**).

The topographic data used in the propagation model came from the BDTOPO^®^ database [22]. Sound levels were predicted for standard environmental conditions (temperature 10 °C, humidity 70%, partially sound absorbing ground) and for eight wind directions (from 0° to 315°, in 45° steps). The sound level assigned to each building corresponds to the sound level in front of the most exposed façade, and to the maximum of the sound levels predicted for the eight wind directions. Two propagation conditions typical of the daytime and night-time periods were also considered. These conditions could be parametrized in the Harmonoise model by choosing classes of meteorological stability adapted to these periods (three classes for day, and two classes for night). For each time period, the class that favored sound propagation was chosen (classes S3 for daytime and S5 for night-time [27]). It is important to note that the assessment of daytime and night-time exposures was not related to the sound a resident would be exposed to during an entire daytime or night-time period (as an equivalent sound level indicator would quantify), but they were related to two different meteorological scenarii that influence the sound propagation differently during these two periods. Sound levels exposures below 30 dBA were excluded as too low to be significant. Finally, the calculations provided the sound level exposure at each building façade in BDTOPO database around all the wind farms. Figure 3 shows an example of the sound radiated from a wind farm (Figure 3a) and the exposure of nearby buildings (Figure 3b) estimated with these calculations.

### 2.4. Count of the Number of People Exposed to WT Sound

Building exposures were cross-referenced using a GIS software (QGIS [28]) with the populations provided by the national database of land use files MAJIC updated in 2016 [29]. Only residential buildings were included in the counting.

Finally, the number of people exposed to WT sound for day- and night-time propagation conditions was determined at the scale of the whole metropolitan France, as well as of the 13 administrative regions in metropolitan France.

### 2.5. Comparison of WT Sound Exposure with Other Environmental Noise Sources

A comparison of WT sound exposure with transportation noise exposure was conducted. Transportation noise exposure data were derived from calculations performed in 2017 [30] as part of the European directive related to the assessment and management of environmental noise [31].

## 3. Results

### 3.1. Evaluation of the Wind Turbine Sound Prediction Model

**Figure 4:**
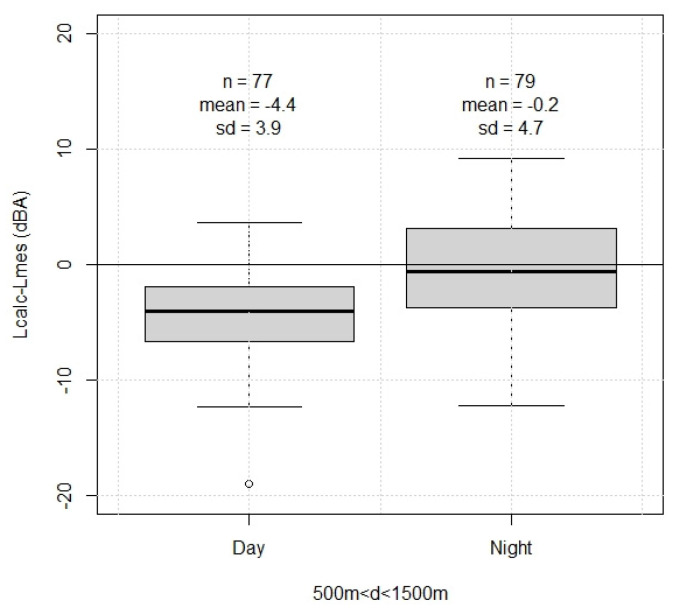
Boxplot of the difference between predicted and measured sound levels, for two typical meteorological conditions during the day and night. The wind speed was above 6 m/s and the distance from the wind turbine ranged from 500 m to 1500 m. Number of samples (n), bias (mean), standard uncertainty (sd).

The distribution of differences between the predicted and measured sound levels was obtained from 77 and 79 measurements where the wind speed was greater than 6 m/s and the distance from the wind turbine ranged from 500 m to 1500 m, for daytime and night-time propagation conditions respectively. The bias was −4.4 dBA and −0.2 dBA, and the standard uncertainty was 3.9 dBA and 4.7 dBA, for daytime and night-time propagation conditions, respectively (Figure 4).

### 3.2. Wind Turbine Sound Exposure in Metropolitan France

The total number of people in metropolitan France exposed to WT sound levels above 30 dBA was 685,770 people for daytime propagation conditions and 721,559 people for night-time conditions (Table 1). Taking into account the standard uncertainty from the model validation resulted in a range of this estimate of [430,036; 905,967] people for daytime meteorological conditions and [303,976; 1,029,390] for night-time. These results corresponded to 1.0% [0.6%; 1.3%] of people living in France in 2017 for daytime, and 1.0% [0.4%; 1.5%] for night-time. It should be noted that the range of these estimates did not correspond to confidence intervals, but to estimates based on the lower and upper scenarii.

**Figure 5:**
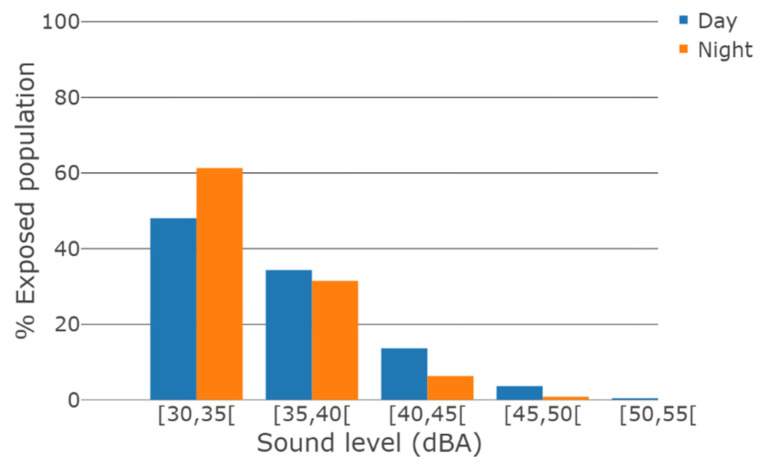
Number of people in metropolitan France exposed to WT sound as a function of WT sound level for daytime and night-time meteorological conditions, normalized by the total number of people exposed to WT sound level above 30 dBA.

Finally, 48% and 61% of the people exposed to sound levels above 30 dBA were exposed to sound levels below 35 dBA for daytime and night-time propagation conditions respectively, and 82% and 93% of the people exposed to sound levels between 30 dBA and 40 dBA for both daytime and night-time propagation conditions respectively (Figure 5).

### 3.3. Wind Turbine Sound Exposure by Region

**Figure 6:**
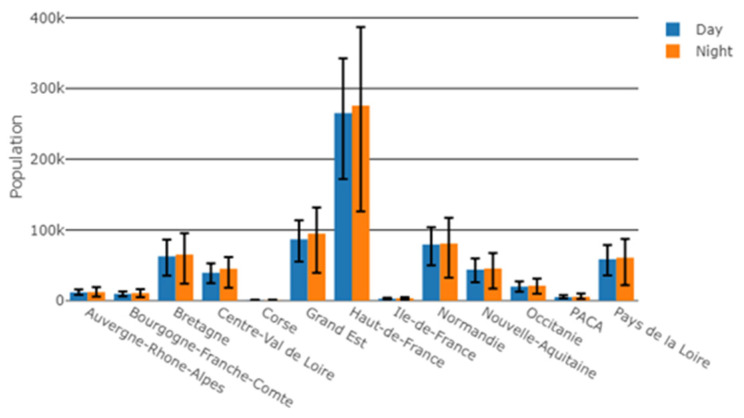
Number of people exposed to WT sound level above 30 dBA, by region. Error bars account for the uncertainty of +/−1 standard deviation on the Harmonoise sound level estimate.

Most of the population exposed to WT sound levels above 30 dBA was located in the Hauts-de-France region (daytime: 265,227 people, night-time: 275,846 people). Bretagne, Grand-Est and Normandie regions accounted for 62,728 to 86,770 people for daytime, and for 65,285 to 94,742 people for night-time (Figure 6).

**Figure 7:**
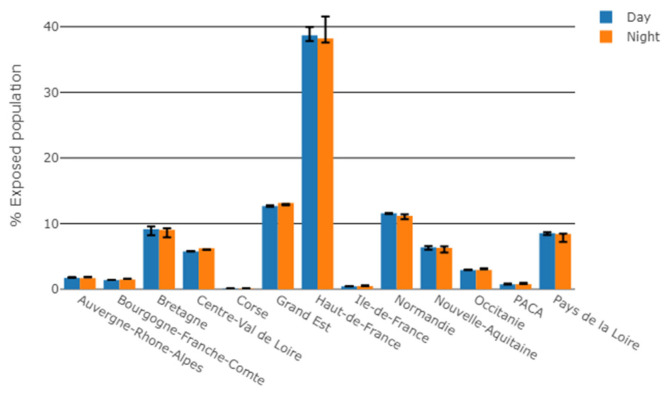
Number of people exposed to WT sound levels above 30 dBA for daytime and night-time meteorological conditions, by region, normalized by the total number of exposed people in metropolitan France. Error bars account for the uncertainty of +/−1 standard deviation on the Harmonoise sound level estimate.

For daytime, 39% of the French population exposed to WT sound levels above 30 dBA lived in Hauts-de-France (38% for night-time) (Figure 7). Bretagne, Grand-Est and Normandie represented 9% to 13% (daytime) and 9% to 13% (night-time) of the population exposed to WT sound levels above 30 dBA.

**Figure 8:**
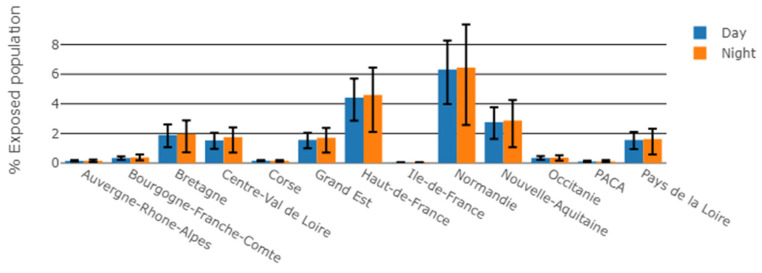
Number of people exposed to WT sound level above 30 dBA for daytime and night-time meteorological conditions, by region, normalized by the total population of each region. Error bars account for the uncertainty of +/−1 standard deviation on the Harmonoise sound level estimate.

Considering the exposed population in relation to the total population of each region, two northern regions had a higher percentage of people exposed to WT sound: Hauts-de-France and Normandie accounted for 4% and 6% for daytime, and 5% and 6% for night-time, respectively, (Figure 8) of the total population of each region.

**Figure 9:**
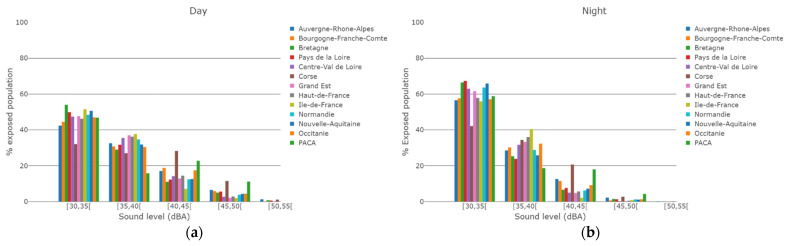
Number of people exposed to WT sound as a function of WT sound levels, normalized by the total number of people exposed to WT sound levels above 30 dBA: daytime (**a**) and night-time (**b**) meteorological conditions.

Figure 9 shows a comparison of the WT sound level distributions of the exposed populations between all regions of France, for both daytime and night-time meteorological conditions. Except for Corse and Provence-Alpes-Côte d’Azur (PACA) regions, for all other regions, the majority of exposed people are exposed to WT sound levels below 35 dBA.

### 3.4. Comparison of WT Sound Exposure with Transportation Noise Exposure

**Figure 10:**
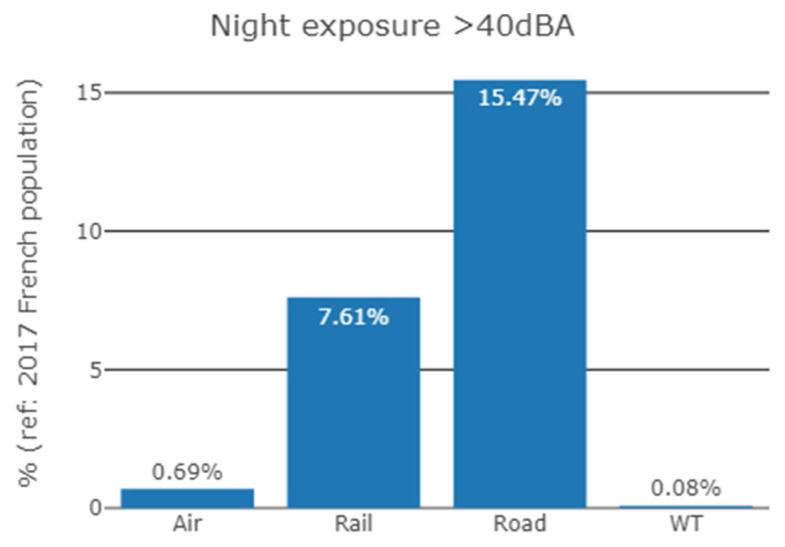
Proportion (%) of the 2017 French population exposed to sound levels above 40 dBA during the night, for four noise sources: aircraft noise (Air), railway noise (Rail), road traffic noise (Road) and wind turbine noise (WT).

Figure 10 shows a comparison of the proportion of people exposed to transportation sound levels above 40 dBA during the night (L_night_) [30] with the population exposed to WT sound levels above 40 dBA for night-time propagation conditions. The French population in 2017 exposed to night-time noise was 10,394,293 for road traffic noise, 5,113,159 for railway noise and 463,611 for aircraft noise, which can be compared to 53,752 people for night-time WT sound exposure; i.e., 15.0%, 7.0%, 0.7% and 0.08% of the 2017 French population, respectively.

## 4. Discussion

The aim of the research presented in this paper was to quantify the number of windfarms’ residents in France exposed to wind turbine sound. The first objective was to validate a sound level prediction model for the calculation of WT sound levels. The performance of the Harmonoise model was evaluated through the quantification of its uncertainties on the WT sound levels predictions. For both daytime and night-time propagation conditions, the bias between predicted and measured WT sound levels was regarded as sufficiently small (bias between −4.4 dBA and −0.2 dBA) to allow a correction of predicted sound levels. The value of the standard uncertainty was considered sufficiently low to validate the use of Harmonoise model for the prediction of WT sound levels in this research. It is consistent with what is encountered for engineering models of outdoor noise prediction, for which the standard uncertainty can typically range from 2 to 4 dBA, depending on the model [12,32,33,34].

The second objective was to count the number of people in metropolitan France exposed to WT sound levels. The proportion of those exposed to WT sound levels above 30 dBA represented a small proportion of the total population in France (1% for both daytime and night-time propagation conditions). The WT sound exposure of these people was very moderate: the majority of people were exposed to sound levels below 35 dBA, and more than 80% were exposed to sound levels below 40 dBA, for both daytime and night-time propagation conditions. Most of the exposed population was located in the Hauts-de-France region (about 40%). A very large majority of the exposed population was located in the North of France: Bretagne, Grand-Est, Normandie and Hauts-de-France represented more than 75% of the people exposed to WT sound.

Compared to other regions, the two northern regions, Hauts-de-France and Normandie have more people exposed to WT sound compared to the total population of each region. Nevertheless, this difference was rather small (less than 5%) and could be partly explained by the combination of several regional factors: the number of wind farms, the number of people living in rural areas, where wind farms were generally installed, and the spatial distribution of people within each region.

The distributions of sound levels between regions were compared in order to investigate regional specificities in the WT sound exposure. This could happen, for example, if WT would be noisier or closer to residents’ dwellings in some regions. Except for Corse and PACA regions, the distributions of sound levels of exposed populations seemed very similar across regions, for both daytime and night-time meteorological conditions. In metropolitan France, people exposed to WT sound were exposed in a similar manner, with no regional specificity. In addition, for almost all regions, the sound exposure of the majority of exposed people was between 30 dBA and 35 dBA. The cases of Corse and PACA were marginal given the small number of exposed people for those regions.

If any significant health effects from WT sound were to be demonstrated in the future, it would be useful to assess this potential public health issue in comparison to other exposures to common noise source for which health effects have already been demonstrated [3]. The comparison of the proportion of the French population in 2017 exposed to sound levels above 40 dBA during the night for transportation noise and for WT sound showed that far few people were exposed to WT sound than to transportation noise (0.08% for WT sound, compared to 15.0%, 7.0%, 0.7% for road traffic noise, aircraft noise and railway noise respectively). This is especially true as the sound levels of the population exposed to transportation noise were underestimated. Indeed, only major transportation infrastructures, and cities with more than 100,000 inhabitants were considered (see [31] for more details). Conversely, WT sound exposure was probably overestimated here (see below). The fact that the proportion of people exposed to WT sound was much lower than that exposed to transportation noise could be explained by the much smaller number of wind farms on the metropolitan territory, compared to transportation infrastructures, by the installation of wind farms in rural areas where the population density is lower, by sound levels at dwellings that are lower for WT, and finally by distances between dwellings and noise sources that are greater for WT due to French legislation about wind farms implantation.

The methodology for assessing the population exposed to WT sound had some limitations. The first limitation, as briefly mentioned above, was that the count of exposed people was likely to be overestimated because the WT sound levels were estimated by considering (i) a constant wind speed corresponding to the nominal operation of the wind turbines, as if WT were constantly in operation throughout the day or night, (ii) the sound exposure based on the maximum sound level among all the sound levels predicted for the different wind directions, (iii) the sound level of the most exposed façade of each dwelling and (iv) the meteorological conditions corresponding to favorable conditions for sound propagation. The initial objective of the paper was to investigate the worst-case scenario that maximized noise exposure levels. Therefore, only one wind speed value was considered (7 m/s at 10 m height). This corresponded to the maximum sound emission of the wind turbine and to the nominal WT operation. It should be noted that the sound emission is constant for wind speeds above 7 m/s. Three scenarii were then considered: the best, the worst and the average scenario, named in the paper lower, upper and average scenario in order to bound the estimates of population counts. The range of estimates could sometimes be wide, but this was not a major problem, the most important being the orders of magnitude of the population counts, and also the values corresponding to the lower scenario, which indicated whether there are enough people exposed to different and relatively contrasting WT sound levels to conduct an epidemiological study and to demonstrate, in a statistically rigorous way, an association between a health condition and the level of wind turbine noise, if such an association exists. In the on-going RIBeolH project [35], information from regional wind statistics will be used to take into account the regional influence of wind direction and speed on exposure. Considering that all inhabitants of a house were assigned to the most exposed façade could also lead to an overestimation of the number of people exposed to the WT sound. This overestimation was not a major concern in this research since one of its original aims was to obtain an upper limit of WT sound exposure. Conversely, if the www.thewindpower.net (accessed on 1 December 2021) database (was the most complete publicly available database of WT in France in 2017, pending the exhaustive database that the French Ministry of the Environment is building and which should be accessible in 2022, its completeness was not perfect and the missing wind farms could be a cause of underestimating the number of residents exposed to WT sound in this study. A comparison of national wind capacity from the database with other data sources [36,37] showed that the underestimation could nevertheless be limited because the database contains 94% of the installed capacity as of mid-2017.

A second limitation was that the predicted sound exposure did not correspond to an actual exposure over a day or night period, as would for example an equivalent noise level, but took into account typical propagation conditions of daytime and night-time periods. However, they could possibly be interpreted as an equivalent sound level exposure if it was assumed that the meteorological conditions and wind speed were the same during all the periods. While this was not the most realistic situation, it nevertheless did provide useful information on the upper limit of the number of people exposed to WT sound.

Another limitation of this research concerned the estimation of uncertainties. Estimating the uncertainties in the population counts (associated with the level of reliability and the percentage of error) was rather difficult because there was no simple relationship between sound exposure and population count: this relationship differed strongly from one wind farm to another due to the propagation influence (e.g., the presence or absence of obstacles), and the spatial distribution of the population around each wind farm. Some procedures based on Monte Carlo or bootstrapping techniques could have been considered, but they were deemed too time-consuming in relation to the time available for this research. They will nevertheless be explored for the update of this research in the framework of the RIBEolH project. Thus, in order to give a range of estimates of the population counts, three noise exposure scenarii (upper scenario, average scenario and upper scenario) were preferred for estimating these uncertainties.

The aim of this study was to deal only with audible noise, which is a first step before going further. It was out of the scope to deal with all phenomena involved in WTN, in particular:

- Tonalities: audible tonalities phenomena than can occur in WT is very often due to a malfunction of the WT (e.g., in the hub machinery). In this study, we have assumed that wind turbines were operating normally.
- Amplitude modulation: nowadays, no available engineering model can model amplitude modulation, and amplitude modulation modeling is still a research challenge (see for example the PIBE project [38], http://www.anr-pibe.fr, (accessed on 1 December 2021). Moreover, the few available research models on amplitude modulation cannot be applied at the scale of a national territory because of their complexity and because they require input information that is not available at this large geographical scale.

The methodology proposed in this paper could be improved: the introduction of annual wind speed and direction statistics for each region in the calculation process could provide a more realistic sound exposure for daytime or night-time periods. An investigation of the low frequency noise exposure could also be done. An update of this work is in progress as part of the preparation of the epidemiological study planned in the RIBEolH project [35], to adapt to the rapid growth in the number of wind farm installations in France and the evolution of the French population, but also to improve the calculation process and to extent the assessment to low frequency noise exposure.

The results presented above, being the first assessment of exposure to wind turbine noise in France, may lead to a relevant epidemiological study in the RIBEolH project. Indeed, in order to conduct an epidemiological study and to demonstrate, in a statistically rigorous way, an association between a health condition and the level of wind turbine noise, if such an association exists, it would first be necessary to recruit a sufficient number of individuals exposed to different and relatively contrasting WT sound levels. It will be possible to reach this first step thanks to the count of the number of people exposed to contrasted levels of WT sound for all wind farms of the whole metropolitan France. Then, the quality of epidemiological studies assessing the risks associated with environmental exposures depends in part on the quality of the estimation or measurement of participants’ exposure. However, in a large-scale epidemiological study, noise measurements in a large number of residents will not be feasible because of the cost and it will be necessary to use noise prediction models. The use of Harmonoise prediction model proposed and validated in this paper will thus make it possible to estimate the exposure to wind noise at the home of each participant who will be included in the epidemiological study of the RIBEolH project.

## 5. Conclusions

The objective of the Cibelius feasibility study was to propose a methodology for calculating WT sound exposure at a national geographical scale and to identify the number of people exposed to WT sound in metropolitan France.

The total number of people exposed to WT sound was approximately 686,000 during the day and 722,000 during the night, thus about 0.1% of the 2017 French population. More than 80% of the population exposed to WT sound levels above 30 dBA was exposed to levels below 40 dBA. It is important to note that, due to some assumptions (wind speed corresponding to WT nominal operation, wind turbines constantly in operation throughout the day), the sound exposure, and therefore the number of people exposed to WT sound, was probably overestimated.

These results constitute the first assessment of WT sound exposure at a national geographical scale, and more specifically for metropolitan France. The results and methodology proposed in this paper were the first step in preparing an epidemiological study in France in the few next years. This study will be conducted in the RIBEolH project, which is investigating the impact of WT sound on human health and annoyance. The results of this study will provide useful information for public authorities to assess whether or not regulations concerning WT should be adapted.

## Figures and Tables

**Table 1:** Number of people in metropolitan France exposed to WT sound levels above 30 dBA for daytime and night-time propagation conditions. The lower/upper scenarii correspond to +/−1 standard deviation on the Harmonoise sound level estimate.

Sound Levels Scenario	Daytime	Night-Time
Average scenario	685,770	721,559
Lower scenario	430,036	303,976
Upper scenario	905,967	1,029,390

## Data Availability

The data for each wind farm is publicly available at www.thewindpower.net, but the complete database was purchased from that site by the authors for this project under a research agreement. The topography and building databases are freely available online (see ref. [22]).
